# Supplementary material for: Research on Multi-Scale Ecological Network Connectivity—Taking the Guangdong–Hong Kong–Macao Greater Bay Area as a Case Study
Source: Int J Environ Res Public Health. 2022 Nov 18;19(22):15268. doi: 10.3390/ijerph192215268 (PMC9690939; doi:10.3390/ijerph192215268)
Supplement: Supplementary file 1 [file ijerph-19-15268-s001.zip › ijerph-1965797-supplementary.pdf]

Supplementary Table S1. List of target species in the study area.

Species Dispersion Scale	Group	Species	Endangerment Category	Habitat Range (ha)
Small scale	10ha 3km	Lepus sinensis	LC	5.93
		Moschus berezovskii Flerov	EN	5–10
Mesoscale	60ha 10km	Manis	CR	66–96
		Macaca assamensis	NT	53–65
		Macaca mulatta	LC	65
		Muntiacus muntjak	LC	100
		Paradoxurus hermaphroditus Pallas	LC	160–1700
Large scale	300ha 30km	Prionailurus bengalensis	LC	300–1270
		Viverricula indica	LC	310
		Paguma larvata	LC	370–590
		Vulpes vulpes	LC	387.34
Extra large scale	600ha 60km	Sus scrofa	LC	500–10000
		Martes flavigula	LC	720
		Paguma larvata	LC	370–590
Ultra-large scale	1000ha 100km	Paradoxurus hermaphroditus Pallas	LC	160–1700
		Prionailurus bengalensis	LC	300–1270
		Sus scrofa	LC	500–10000

Note: The endangered grades LC, NT, VU, EN, and CR represent five levels of least concern, imminent threat, vulnerable, endangered, and critically endangered, respectively.

Supplementary Table S2. Ecological sources, potential corridors and average corridor lengths at five dispersion scales from 1990 to 2020.

Years	Index	Small Scale	Mesoscale	Large Scale	Extra Large Scale	Ultra-Large Scale
1990	Ecological source/(Number)	2565	725	265	190	131
	Potential Corridor/(Number)	2133	724	327	287	222
	Average corridor length/(m)	219.99	485.08	1178.12	2465.06	3680.04
2000	Ecological source/(Number)	2626	771	269	199	138
	Potential Corridor/(Number)	2006	770	308	282	209
	Average corridor	206.93	455.81	1075.71	2139.77	3210.69

	length/(m)					
2010	Ecological source/(Number)	2801	778	269	189	119
	Potential Corridor/(Number)	2171	768	310	206	181
	Average corridor length/(m)	194.19	392.99	1034.09	2163.13	3191.59
2020	Ecological source/(Number)	2993	845	291	199	130
	Potential Corridor/(Number)	2416	866	343	288	203
	Average corridor length/(m)	195.72	397.55	929.23	2027.02	2885.28

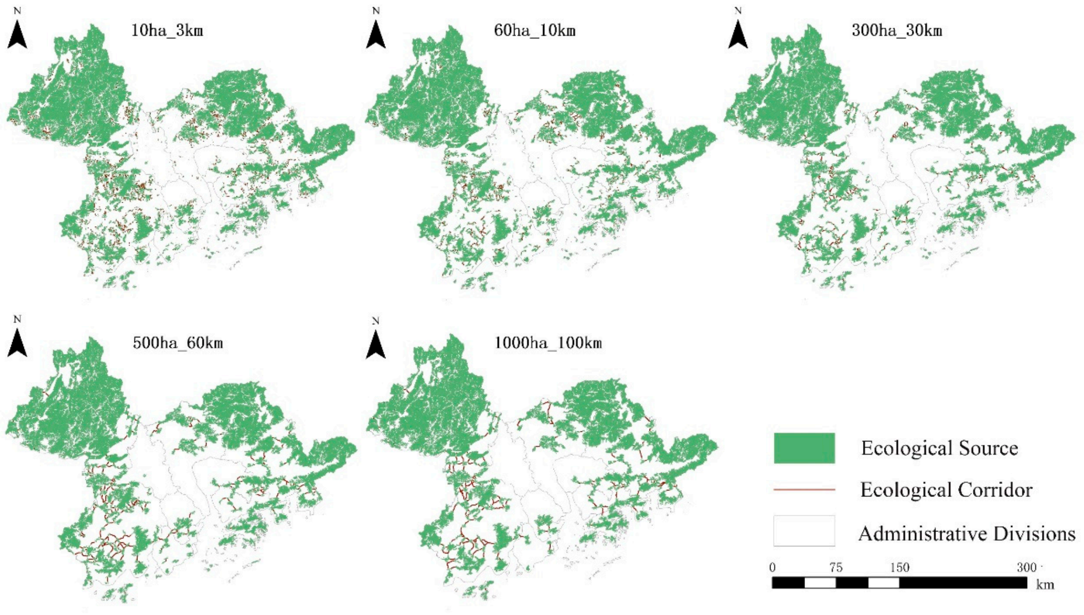

Supplementary Figure S1. Five Scattered Scale Ecological Networks in the Guangdong-Hong Kong-Macao Greater Bay Area in 2000.

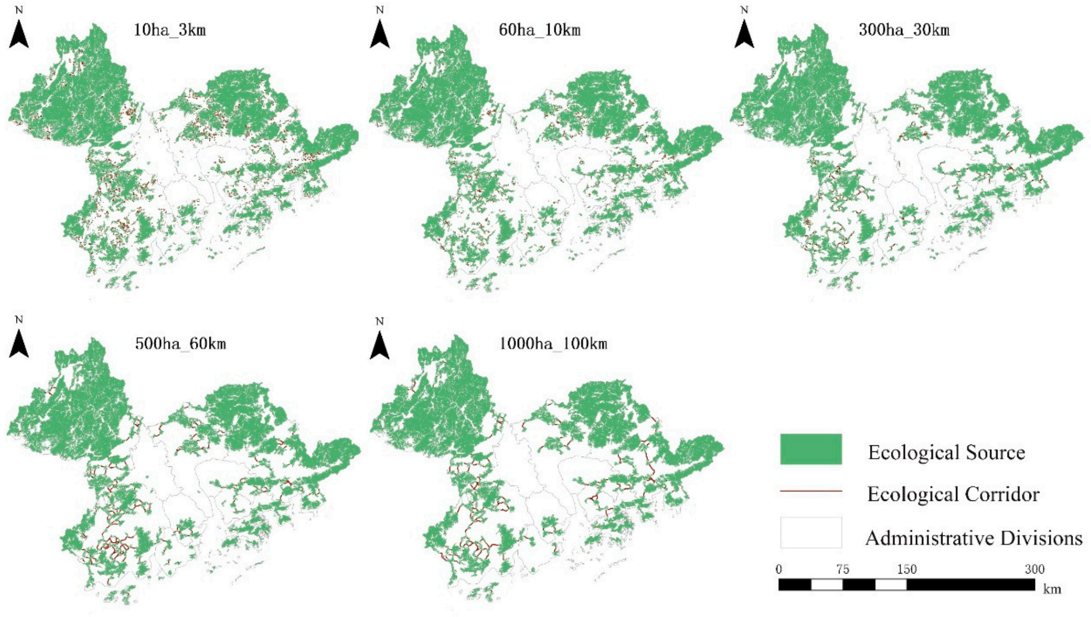

Supplementary Figure S2. Five Scattered Scale Ecological Networks in the Guangdong-Hong Kong-Macao Greater Bay Area in 2010.
